# Bücher am Bildschirm: Empirische Befunde zu Leseerfahrungen und Lesepraktiken mit digitalisierter Literatur

**DOI:** 10.1007/s41244-023-00294-2

**Published:** 2023-08-31

**Authors:** Lukas Kosch, Günther Stocker, Annika Schwabe, Hajo G. Boomgaarden

**Affiliations:** 1 Institut für Germanistik, Universität Wien, Wien, Österreich; 2 Institut für Publizistik- und Kommunikationswissenschaft, Universität Wien, Wien, Österreich

**Keywords:** Gedruckte vs. digitale Bücher, Digitales Lesen, E‑Books, Empirische Leseforschung, Lesepraktiken, Leseerfahrung, Print vs. Digital Books, Digital Reading, E‑Books, Empirical Reading Research, Reading Practices, Reading Experience

## Abstract

Mit E‑Readern, Smartphones, Notebooks und Tablets sind neue Lesemedien entstanden, deren Haptik, Räumlichkeit, Visualität und Materialität sich grundlegend von denen des traditionellen Buches unterscheiden und die von einer Reihe unterschiedlicher Textdarstellungen und damit verbundenen Nutzungshandlungen gekennzeichnet sind. Die Frage nach den konkreten Praktiken des literarischen Lesens am Bildschirm und den damit verbundenen literarischen Erfahrungen wird in diesem Beitrag auf der Basis eines Laborexperiments (*N*=207), einer Fokusgruppenstudie (*N*=34) und einer quotenbasierten Online-Umfrage (*N*=779) beantwortet. Die synoptische Auswertung dieser drei veröffentlichten Studien zeigt, dass gerade eine praxeologische Perspektive wichtige Erkenntnisse für das Verständnis der Unterschiede zwischen dem Lesen gedruckter Bücher und E‑Books erbringen kann. Sowohl was die Menge des Gelesenen betrifft als auch die Lektüreauswahl, die Leseorte und Lesesituationen sowie die Erwerbs- und Aufbewahrungsformen, münden die unterschiedlichen Materialitätsprofile von gedruckten und digitalisierten Büchern in unterschiedlichen Praxeographien. Beide Lesemedien erfüllen unterschiedliche Funktionen, gehen mit verschiedenen Lesepraktiken einher und ergänzen sich vielmehr als sich zu ersetzen.

## Abstract (en)

With e‑readers, smartphones, notebooks, and tablets, new reading media have emerged whose haptics, spatiality, visuality, and materiality differ fundamentally from those of the traditional book. Electronic reading devices are characterized by a range of different text representations and distinct associated reading practices. This article will address the question of the concrete practices of literary reading on screens and the specific literary reading experiences on the basis of a laboratory experiment (*N*=207), a focus group study (*N*=34), and a quota-based online survey (*N*=779). The synoptic evaluation of these three published studies shows that a praxeological perspective in particular can yield important insights for understanding the differences between reading printed books and e‑books. The different material conditions of digitized and printed books result in different practices, both in terms of the quantity of what is read and the choice of text, the reading locations and reading situations, and the forms of acquisition and storage. However, both reading media fulfill different functions, go hand in hand with different reading practices, and complement rather than replace each other.

## Einleitung

Spätestens seit den Arbeiten von Friedrich Kittler und dem von Hans Ulrich Gumbrecht und K. Ludwig Pfeiffer herausgegebenen Sammelband mit dem programmatischen Titel *Materialität der Kommunikation* (1988) ist die Frage der medialen Gestalt und der materiellen Grundlagen von Texten auch in der Literaturwissenschaft angekommen. Dies lässt sich ebenso mit Rückgriff auf Walter Benjamins Kunstwerk-Aufsatz oder Marshall McLuhans Theorie-Slogan (›The medium is the message‹) aufzeigen wie mit Blick auf die Arbeiten der historischen Buchwissenschaften, etwa bei Roger Chartier, oder der *Sociology of Texts* nach D.F. McKenzie. Die Materialität der Textmedien beeinflusst Produktion, Distribution und Rezeption von Texten in bedeutsamer Weise, wie sich in der Mediengeschichte der Literatur zeigt. Régis Debray formuliert pointiert: »Die Historiker des Geschriebenen wissen […], dass die Geschichte der Zeichen mit der Geschichte der Materialien beginnt. […] Es gibt keine unschuldigen Träger, jedes Material fordert seinen Preis.«^1^ Dieser ›Preis‹ blieb in der Literaturwissenschaft lange Zeit unbeachtet, da die Dominanz des gedruckten Buches in den vergangenen Jahrhunderten es gleichsam zum natürlichen Träger literarischer Texte gemacht hat. Seine Medialität und seine materiellen Grundlagen sind dabei weitgehend unsichtbar geblieben. Lesende werden jedoch nie mit abstrakten, aller Dinglichkeit enthobenen Texten konfrontiert, die ja ohne ihre Trägermedien nicht existieren, sondern »sie gehen mit Objekten um, deren Organisation eine Lektüre vorgibt, welche wiederum das Erfassen und Verstehen des gelesenen Textes bestimmt«^2^, auch wenn es sich dabei eher um ein Spektrum möglicher Lektüreweisen handelt als um eine eindeutige und zwangsläufige Koppelung.

Mit E‑Readern, Smartphones, Notebooks und Tablets sind in den letzten vier Jahrzehnten neue Lesemedien entstanden, deren Haptik, Räumlichkeit, Visualität und Materialität sich grundlegend von denen des traditionellen Buches unterscheiden und die von einer Reihe unterschiedlicher Textdarstellungen und damit verbundenen Nutzungshandlungen gekennzeichnet sind.^3^ Harun Maye verweist darauf, dass es eine sehr eindimensionale Vorstellung sei, »das Buch als ›Fortschritt‹ gegenüber der Schriftrolle und den PC oder E‑Reader wiederum als ›Fortschritt‹ gegenüber dem Buch zu bezeichnen, denn nicht nur ermöglicht jedes Medium neue Gebrauchsweisen, sondern beschränkt gleichzeitig auch ältere und andere Formen des Umgangs mit Texten.«^4^ Aus einer medienbewussten literaturwissenschaftlichen Perspektive ist daher die Frage zu stellen, welche Konsequenzen die Digitalisierung von literarischen Texten und das damit verbundene Lesen von Literatur auf Bildschirmen für den Leseprozess und die Leseerfahrung hat und welche konkreten Lektürepraktiken damit einhergehen. Was Julika Griem retrospektiv mit Blick auf die Lesegeschichte formuliert, lässt sich auch für das Verständnis der gegenwärtigen Transformationen behaupten: »Wenn wir mehr über die historischen Praktiken des Lesens erfahren wollen, reicht es nicht, die Inhalte von Texten und ihre Aussagen und Deutungsangebote zu studieren. Wir müssen uns mit der Widerständigkeit und Obsoleszenz der Schriftträger, mit der Geschichte und damit auch mit der Rekonstruierbarkeit von Textualität wie von Lesepraktiken befassen.«^5^

Lange Zeit wurde die digitale Revolution von Seiten der Kultur-und Geisteswissenschaften im Signum der Krise diskutiert. Von Sven Birkerts *The Gutenberg Elegies. The Fate of Reading in an Electronic Age* (1994) über Nicholas Carrs *The Shallows – What the Internet Is Doing to Our Brains* (2010) bis zu Maryanne Wolfs *Reader Come Home. The Reading Brain in a Digital World* (2018) – um nur einige der international einflussreichsten Titel zu nennen – wurde vor den angeblich überwiegend negativen Folgen der Digitalisierung für das Lesen gewarnt. Die Verbreitung digitaler Lesegeräte, die Popularisierung digitaler Texte und das digitale Lesen per se werden dabei als Bedrohung für die literarische Kultur diskutiert bzw. mit einem quantitativen und qualitativen Rückgang des Lesens in Verbindung gebracht. Mit einem kulturpessimistischen Impetus wird auch in der Öffentlichkeit immer wieder breit diskutiert, dass die Konzentration auf längere, narrative Texte und die Fähigkeit zum ›deep reading‹ durch die allgegenwärtige Nutzung digitaler Medien verloren gehe^6^ und dass Lesende digitaler Texte leicht abgelenkt werden und Texte auf Bildschirmen eher oberflächlich als gründlich lesen.^7^

Diese Art der Kulturkritik lässt sich als Ausdruck der Verunsicherung verstehen, die durch die rasche Verbreitung digitaler (Lese‑)Medien ausgelöst wurde. Allerdings, wie Gerhard Lauer zu Recht konstatiert, »steht der Sicherheit im Urteil über den Stand des Lesens eine vergleichsweise schmale Datenbasis gegenüber, die aufschlüsselt, was es mit dem Lesen auf sich hat, seit es Computer und Internet gibt.«^8^ Zwar sind die Folgen der digitalen Revolution für die Produktion und den Vertrieb von Literatur bereits ausführlich erörtert worden,^9^ aber die Konsequenzen für das literarische Lesen und insbesondere für die Lesenden bilden nach wie vor ein Forschungsdesiderat.^10^ Insbesondere E‑Books gelten für ihre Nutzer:innen nicht mehr als technische Innovation, sondern sind fest in Mediennutzungsroutinen implementiert und erfordern daher eine Untersuchung, die von der technischen und materiellen Beschaffenheit der Textträger ausgeht und auf die damit verbundenen Gebrauchsweisen fokussiert. Denn die Buchlesenden stehen heute mehr denn je vor der Auswahlmöglichkeit, ob ein konkreter literarischer Text gedruckt oder digital gelesen bzw. als Hörbuch rezipiert wird.^11^ Während die empirische Leseforschung auf die Herausforderung der Digitalisierung reagiert hat und seit einiger Zeit versucht, mittels verschiedener Experimente Daten über konkrete Leseprozesse zu gewinnen (wobei allerdings das Lesen von literarischen Texten seltener untersucht wird), so hat sich die Literaturwissenschaft bisher nur wenig damit auseinandergesetzt. In ihrem 2018 erschienenen umfassenden literaturwissenschaftlichen Handbuch zum Thema Lesen konstatieren Alexander Honold und Rolf Parr, »dass in den neuphilologischen Literaturwissenschaften die Fragen nach Techniken, Formen und Gegenständen des Lesens, gemessen an ihrer strategischen Relevanz, weiterhin (oder wieder) eine nur nachrangige Rolle zu spielen scheinen.«^12^

Angesichts der Aktualität des Themas und der in mehrerlei Hinsicht unbefriedigenden Forschungslage wollen wir in diesem Artikel die Frage nach den konkreten Praktiken des literarischen Lesens am Bildschirm und den damit verbundenen literarischen Erfahrungen auf der Basis eines Laborexperiments (*N*=207), einer Fokusgruppenstudie (*N*=34) und einer quotenbasierten Online-Umfrage (*N*=779) beantworten – als Beitrag zu einer »medienbewussten Philologie«, wie sie Niels Penke und Niels Werber in ihrer Einleitung zu dem einschlägigen Themenheft der *Zeitschrift für Literaturwissenschaft und Linguistik* gefordert haben.^13^ Es handelt sich im Folgenden um die Zusammenführung von drei bereits veröffentlichten Studien, deren wichtigsten Ergebnisse zu den Leseerfahrungen und Lesepraktiken mit digitalisierter Literatur nun in einer synoptischen Auswertung und theoretischen Rahmung präsentiert werden.^14^

## Theoretische Prämissen

Ist es das Ziel, zu untersuchen, was Menschen in verschiedenen Situationen und Kontexten mit Medien tun, so kann an theoretische Ansätze angeschlossen werden, die materielle Objekte aus einer praxeologischen Perspektive in den Blick nehmen.^15^ In Anlehnung an Bruno Latour und Theodore R. Schatzki definiert Andreas Reckwitz das Materielle als Ansammlung von Artefakten oder hybriden Dingen, »die analog zu den menschlichen Akteuren an den sozialen Praktiken notwendigerweise partizipieren.«^16^ Im Sinne einer daraus resultierenden Praxistheorie werden Gegenstände nicht darauf reduziert, dass sie auf eine spezifische Weise interpretiert werden, sondern es wird der Fokus vor allem darauf gelegt, auf welche Weise sie gehandhabt werden und welche spezifischen Praktiken sie ermöglichen: »Die Materialität der Artefakte beeinflusst (aber determiniert nicht), welches praktische Verstehen und folglich welche sozialen Praktiken möglich sind.«^17^

Seit einigen Jahren werden praxeologische Ansätze vermehrt in den historisch ausgerichteten Philologien in den Blick genommen, um die Bedeutung der Materialität des Geschriebenen für soziale Praktiken zu betonen und insbesondere um wahrscheinliche Rezeptionsszenarien zu entwerfen. In Analogie dazu können die Prämissen der materialen Textkulturforschung und Text-Anthropologie^18^ auch für gegenwärtige Rezeptionsszenarien übernommen werden. Die drei Bereiche, die Markus Hilgert als Darstellungsformate einer praxeologisch perspektivierten Artefaktanalyse definiert, werden als »Materialitätsprofile«, »Topologien« und »Praxeographien« bezeichnet.^19^ Für die Analyse der zeitgenössischen Rezeption von literarischen Texten auf verschiedenen Lesemedien erweist sich die Terminologie der Materialitätsprofile und Praxeographien als besonders anschlussfähig. Dokumentieren Materialitätsprofile die Gesamtheit der stofflichen Charakteristika von Artefakten, so beziehen sich Praxeographien auf das »reziproke Verhältnis zwischen schrifttragendem Artefakt und Körpern, zwischen material präsentem Geschriebenen und den daran tatsächlich oder wahrscheinlich vollzogenen Rezeptionspraktiken«.^20^

Gilt es Materialitätsprofile für das gedruckte Buch und die entsprechende digitalisierte Version zu erstellen, so sind die medientechnischen Unterschiede offensichtlich. Aus dieser Perspektive hat das E‑Book nur wenig mit einem gedruckten Buch gemeinsam. Um nur drei der wichtigsten Unterschiede zu nennen:

- Elektronische Texte existieren an keinem festen Ort. Die für das gedruckte Buch bestimmende Einheit von Speichern, Prozessieren und Übertragen ist aufgelöst.^21^ Diese Trennung von Speichermedium und Lesemedium ist ein medienhistorisch bedeutsamer Schritt mit weitreichenden Konsequenzen für die Distribution, die Zugänglichkeit, die Kontrollierbarkeit und die Dauer von Texten,^22^ aber auch für die Textauswahl und den Lektüreprozess. Denn das gedruckte Buch ist ausschließlich Textträger eines bestimmten literarischen Werkes, wohingegen elektronische Lesegeräte multimodale und multifunktionale Objekte sind, die verschiedene Formen der Interaktion und Aufmerksamkeit, aber auch einen raschen Wechsel von einem Buch zu einem anderen erlauben.^23^ Andererseits kann ein und dasselbe E‑Book auch auf mehreren verschiedenen Endgeräten wie Smartphone, Tablet, Notebook oder E‑Reader gelesen werden.
- Materielle Bücher sind dreidimensionale Objekte in Form »gebundener und in Lagen geschichteter Seiten«, sie ordnen »Schriftzeichen nicht nur flach, sondern auch räumlich.«^24^ Demgegenüber sind elektronische Lesegeräte durch ihre zweidimensionalen Bildschirme charakterisiert, die von außen nicht erkennen lassen, wieviel Text vor oder nach der gerade aufgerufenen Seite vorhanden ist. Indem die Buchstaben aber bewegt und in ihrer Größe verändert werden können, variieren die Angaben zu den verbleibenden Seiten aufgrund der individuellen Einstellungen.
- Digitale Texte haben kein materielles Substrat und sind damit höchst flexibel, d. h. von Plattform zu Plattform beweglich und veränderbar. Demgegenüber steht die räumliche und zeitliche Permanenz gedruckter Texte, die mit anderen senso-motorischen Bedingungen und Möglichkeiten einhergeht.^25^ Roland Reuß hat in einem pointierten Essay die Bedeutung des gedruckten Buches und seiner Typographie als ideale Anordnung des Satzmaterials, »in dem komplexe Gedanken *ihre* Gestalt, und d. h. ihre angemessene Gestaltung, finden«,^26^ der »typographischen Obdachlosigkeit von Schrift im Netz«^27^ und den »im Kern antitypographisch(en)«^28^ digitalen Medien gegenübergestellt. Man muss Reuß’ Wertungen nicht in ihrer zum Teil polemischen Zuspitzung übernehmen, trotzdem trifft seine detailgenaue Analyse der *Ergonomie des Buches* grundlegende mediale Differenzen zwischen papierenen und elektronischen Büchern. Der offensichtliche Vorteil der Flüssigkeit und Flüchtigkeit digitaler Medien gegenüber der stabilen Drucktechnik ist andererseits die vielfältige Möglichkeit zur individuellen Anpassung und zur Interaktivität mit Texten, die einfacher geteilt, bearbeitet und aufgerufen werden können.^29^

Der von James J. Gibson geprägte und in der neueren empirischen Leseforschung breit angewendete Begriff der Affordanz^30^, der den Angebotscharakter eines Objekts bezeichnet,^31^ erlaubt es über die bloße Beschreibung der technischen Unterschiede hinaus zu gelangen. Die materielle Gestaltung des gedruckten Buches oder digitaler Lesegeräte und deren Leseapplikationen, d. h. die physischen Eigenschaften des Gegenstandes, geben demnach spezifische Gesten und Techniken der Buchnutzung vor. In diesem Sinne gehen Materialitätsprofile auch der Frage nach, welche Handlungen und Praktiken die materialen Eigenschaften eines Artefakts ermöglichen oder verhindern.^32^ Die spezifischen, inhärenten Eigenschaften und Affordanzen von gedruckten Büchern und elektronischen Lesegeräten erfordern eine unterschiedliche Beteiligung des motorischen Systems an der Verarbeitung von Texten und unterschiedliche sensomotorische Aktivitäten, denn »the haptic feedback of a touch screen is different from a paper book, and the implications of such interactions warrant empirical investigations«.^33^

In der vorliegenden Forschung wird am häufigsten die Technik des Blätterns diskutiert, sowohl in medialer als auch in historischer Perspektive. Augenscheinlich drängt sich die Tatsache auf, dass die blätternde Lektüre dem Medium Buch vorbehalten bleibt, »denn die alten Schriftrollen und die neuen Bildschirme lassen sich nicht durchblättern, sondern nur abrollen, umschalten oder wischen.«^34^ Wird der Leseprozess in diesem Sinne als verkörperte Aktivität aufgefasst, die über die kognitive Tätigkeit hinausgeht und in die Augen, Hände und Körperhaltung involviert sind, so liegt die Annahme nahe, dass unterschiedliche Benutzeroberflächen und unterschiedliche dispositionelle Eigenschaften des Lesegeräts in unterschiedlichen Praxeographien kulminieren.^35^

Für Praxeographien sind die Darstellungen von routinierten Praktiken zentral, und es ist der Frage nachzugehen, welche Handlungsmöglichkeiten und Handlungsbegrenzungen mit einem Artefakt verbunden sind.^36^ Michael Burke weist darauf hin, dass eine wichtige Phase des Leseprozesses bereits vor dem ersten unmittelbaren Augenkontakt mit dem Text stattfindet.^37^ Denn bevor mit dem literarischen Lesen begonnen wird, muss die Entscheidung getroffen werden, *wo, wann, wie* und *warum* die Lektüre *welches* bestimmten Textes stattfinden soll. Die mit dem Lesen von Literatur verbundenen und zu untersuchenden Praktiken reichen daher von der Textauswahl und dem Bucherwerb über die Wahl der Leseorte und Lesezeiten sowie über die Navigation durch den Text und die konkrete Leseerfahrung bis zur möglichen Anschlusskommunikation und gegebenenfalls dem Aufbewahren des materiellen oder elektronischen Buches. Im Folgenden soll gezeigt werden, dass gerade eine praxeologische Perspektive wichtige Erkenntnisse für das Verständnis der Unterschiede zwischen dem Lesen gedruckter und digitalisierter Literatur erbringen kann.

## Zur Methodik

Obwohl sich die empirische Leseforschung seit geraumer Zeit ausführlich mit der Lektüre auf Bildschirmen und mittels elektronischer Lesegeräte beschäftigt, spielt die Frage nach dem Lesen literarischer Texte dabei eine untergeordnete Rolle. Während in einer Reihe von Metastudien, die das Lesen von Informationstexten betreffen, klar messbare Unterschiede bezüglich Leseleistung und Textverständnis zwischen Texten am Bildschirm und in gedruckter Form festgestellt wurden, konnte ein solcher »print advantage« bei den wenigen Studien zu narrativen Texten nicht verzeichnet werden.^38^ Das mag – neben der prinzipiell eher geringen Anzahl an Studien – auch an den aus literaturwissenschaftlicher Sicht oft problematischen Untersuchungsanlagen liegen.^39^ Für unsere Fragestellung erschien daher eine Kombination aus quantitativen und qualitativen Methoden am zielführendsten, in welcher die sozialwissenschaftliche Empirik mit explizit literaturwissenschaftlichen Kategorien und Perspektiven ergänzt und präzisiert wurde.

Die im Folgenden präsentierten Ergebnisse basieren auf drei empirischen Studien, die wir zwischen 2019 und 2022 durchgeführt haben. Dabei handelte es sich erstens um ein Laborexperiment, bei dem *N*=207 Proband:innen im Alter zwischen 19 und 72 Jahren (Durchschnitt 29,96 Jahre; *n*=168 weiblich, *n*=38 männlich, *n*=1 nicht-binär) die ersten 20 Seiten eines modernen Romans lasen, nämlich Arno Geigers Roman *Schöne Freunde*^40^. Die Hälfte der Teilnehmer:innen las den Text in der Hardcoverausgabe, die andere Hälfte auf einem E‑Reader der Marke Kindle Paperwhite mit einem 6‑Zoll 300dpi-Display (»between-subjects-design«). Da wir nach expliziten Buchleser:innen suchten, wurden die Teilnehmer:innen über Aushänge in Buchhandlungen, Bibliotheken, Theatern und über Social Media eingeladen, an dem Experiment teilzunehmen. Vor und nach der Lektüre füllten sie einen umfangreichen Fragebogen aus und es konnte festgestellt werden, dass niemand von ihnen den Text zuvor gelesen hatte. Zur Messung der kognitiven und emotionalen Leseerfahrung wurde die »Aspekte des Leseerlebens«-Skala von Appel, Koch, Schreier und Groeben herangezogen, die mit insgesamt 77 Items auf 14 Faktoren abzielt, von »Aufmerksamkeitsfokussierung« über »Vorstellbarkeit« und »Räumliches Dabeisein« bis zu »Mitleid«, »Identifikation« und »Analysierende Rezeption«.^41^ Des Weiteren wurde Koopmans »Narrative and Aesthetic Feelings«-Skala verwendet, die Faktoren wie Sympathie/Empathie, Identifikation, Absorption, Mitleid, Attraktivität und Foregrounding untersucht.^42^ Von uns selbst entwickelt wurde eine Fragebatterie, die detailliert auf die Inhalte, die Erzählstruktur und die stilistischen Besonderheiten des ausgewählten Romans eingeht. Die Messung der Lesezeit und eine Erhebung der soziodemographischen Daten der Teilnehmer:innen ergänzten das experimentelle Setting. Die erhaltenen Daten wurden mit einer Reihe statistischer Analyseverfahren ausgewertet.^43^

Zur Vertiefung, Erweiterung und Ergänzung der Ergebnisse des Experiments führten wir zweitens eine Fokusgruppenstudie mit insgesamt *N*=34 regelmäßigen Leser:innen von E‑Books durch (*n*=26 Frauen, *n*=8 Männer im Alter zwischen 18 und 57 Jahren), die auf die gleiche Weise wie die Teilnehmer:innen des Experiments gewonnen wurden. In sechs Gruppen wurden ca. 90-minütige semistrukturierte Interviews geführt, mit Video aufgezeichnet, transkribiert und mit der qualitativen Datenanalysesoftware MAXQDA codiert und anschließend ausgewertet.^44^ Im Zentrum standen dabei Fragen nach der Motivation für das Lesen von E‑Books, nach konkreten Lesepraktiken, Lesesituationen, nach der Genreauswahl und den medienspezifischen Leseerfahrungen.

Drittens führten wir eine quotenbasierte Online-Umfrage^45^ durch, die sich ebenfalls an erwachsene Buchleser:innen richtete. Unser Sample bestand aus *N*=779 Personen (*n*=418 Frauen, *n*=361 Männern) im Alter zwischen 18 und 82 Jahren (Durchschnittsalter 48,69 Jahre), die angaben, regelmäßig Bücher in ihrer Freizeit zu lesen. 47,5 % davon lasen ausschließlich gedruckte Bücher, 43,3 % gedruckte Bücher und E‑Books und 9,2 % ausschließlich E‑Books. Von den E‑Book-Leser:innen verwendeten 34,9 % einen E‑Reader, 16 % ein Tablet, 15,8 % ein Smartphone und 10,1 % einen Computer oder ein Notebook. Der Fokus der Umfrage lag darauf, welche Auswirkungen die Digitalisierung auf das Freizeitleseverhalten verschiedener soziodemographischer Gruppen (Alter, Bildung etc.) hat. In Fortsetzung der Ergebnisse der Fokusgruppeninterviews ging es insbesondere um die Frage nach Genreauswahl, Leseorten, Lesesituationen und den Umfang der gelesenen Bücher jeweils im Verhältnis zur spezifischen Wahl eines Lesemediums. Denn ein wesentlicher Effekt der Digitalisierung von Literatur ist die Tatsache, dass Buchleser:innen zwischen verschiedenen Lesemedien für den gleichen Text auswählen können oder vielmehr müssen und dass sie das auch sehr bewusst tun. Die erhobenen Daten wurden wiederum mit standardisierten statistischen Verfahren analysiert und ausgewertet.^46^

## Zentrale Ergebnisse

Ausgehend von der Frage nach den Konsequenzen der Digitalisierung von Literatur für zeitgenössische Lesepraktiken und Leseerfahrungen lassen sich die Ergebnisse unserer empirischen Studien wie folgt zusammenfassen:

### Leseerfahrung und Textverstehen

Obwohl wir unser Laborexperiment mit größtmöglicher externer Validität durchgeführt haben (gebundenes Buch und verbreiteter E‑Reader, längerer Lesetext von 20 Seiten mit einer durchschnittlichen Lesezeit von 28.31 Minuten, keine technischen Geräte zu physiologischen Messungen, eine möglichst natürliche Leseumgebung mit angenehmem Licht und Ohrensessel) und wir die Messinstrumente differenziert auf den konkreten literarischen Text abgestimmt haben, vor allem was inhaltliche Feinheiten und erzählerische wie stilistische Besonderheiten betrifft, konnten wir keine messbaren Unterschiede bei der Lektüre des Textes aufgrund des Lesemediums feststellen. Weder was das Textverständnis noch was spezifisch literarische Leseerfahrungen wie Immersion, Identifikation oder Empathie betrifft, konnten signifikante Unterschiede zwischen den Gruppen, die den Beginn des Romans in der gedruckten Ausgabe oder am Kindle E‑Reader gelesen hatten, gemessen werden. Einzig bei der Rekonstruktion von Textinhalten, die am Beginn des gelesenen Abschnitts angesiedelt waren, schnitten die Print-Leser:innen etwas besser ab. Am Anfang des Lektüreprozesses scheint die dreidimensionale Räumlichkeit des Buchkörpers die Orientierung im Text und die Erinnerungsleistung zu unterstützen.

Eine mögliche Erklärung für die geringen Unterschiede in der Leseerfahrung könnte in der spezifischen Wirkungsweise aller Medien gefunden werden. So verweist Sybille Krämer auf deren grundlegende immersive Tendenz, denn »insoweit Medien in ihrem Gebrauch reibungslos, mithin störungsfrei funktionieren, tendieren sie dazu, etwas zur Erscheinung zu bringen, indem sie selbst sich dabei zurücknehmen und in ihrer eigenen Materialität und Verfasstheit unterhalb der Schwelle des Wahrnehmens verbleiben.«^47^ In der Funktion, Abwesendes zur Darstellung zu bringen, »werden Objekte also gerade nicht hinsichtlich ihrer präsenten Materialität betrachtet, sondern fungieren als durchlässiger Kanal für eine medial vermittelte Botschaft.«^48^ Dies gilt insbesondere für den konkreten Akt des Lesens, denn es ist der Schrift eigen, dass nicht die materielle Manifestation des Textträgers im Vordergrund der Rezeption steht, vielmehr muss sie in den Hintergrund treten, um eine reibungslose und immersive Lektüre zu ermöglichen: »Nur weil die Schrift zulässt, dass der Blick des Lesers nicht um ihrer selbst willen an ihr haften bleibt, sondern über sie hinweg und durch sie hindurchgeht, findet Lesen statt, verwandelt sich die Sinnlichkeit der Buchstaben in Sinn.«^49^ Verbirgt sich ein Medium im Prozess seiner Mediatisierung^50^, so gilt das insbesondere für narrative Texte, die eine besonders starke immersive Wirkung haben können.

Darüber hinaus hat Rolf A. Zwaan in seinen Studien zeigen können, dass allein die Annahme, einen literarischen Text zu lesen, ein spezifisches kognitives Kontrollsystem auslöst, welches das Verstehen des Textes regelt.^51^ Selbst wenn derselbe Text gelesen wird, gibt es Unterschiede in der neuronalen Verarbeitung, je nachdem, ob die Lesenden davon ausgehen, einen Informations- oder einen literarischen Text zu lesen.^52^ Bei der Lektüre eines literarischen Textes ist für die Lesenden nicht von Beginn an erkennbar, welche Informationselemente noch wichtig sein werden. Die Bedeutungsstruktur entfaltet sich erst sukzessive während des Leseprozesses und der Text erfordert daher eine ganz spezifische Aufmerksamkeit bezüglich der Textbasis und Oberflächenstruktur und ein gutes Gedächtnis für wörtliche Informationen.^53^ Es ist naheliegend, dass diese stärker auf den Text ausgerichtete Lesestrategie beim Lesen von Erzählungen dazu führt, dass das Lesemedium an Bedeutung verliert.

Während wir also im konkreten Akt des Lesens kaum medienspezifische Unterschiede finden konnten, ließen sich jedoch solche auf der Ebene der Lesepraktiken und der Umgangsformen mit den konkreten Lesemedien durchaus erkennen, wie wir in unserer Fokusgruppenstudie und der Online-Befragung feststellen konnten. Bezüglich der Frage nach den Konsequenzen der Digitalisierung des Bücherlesens ist es daher notwendig, den Blick vom Leseprozess auf das Nutzungsverhalten im Umgang mit gedruckten und digitalen Büchern zu lenken.

### Lesemenge und Lektüreauswahl

Es ist ein Gemeinplatz in der Diskussion über die Konsequenzen der Digitalisierung geworden, zu beklagen, dass immer weniger Menschen Bücher lesen würden. Gerhard Lauer wendet sich jedoch gegen kulturpessimistische Verfallsthesen und konstatiert, dass heute »mehr Bücher denn je verkauft und so intensiv und selbstversunken wie früher verschlungen« werden.^54^ Unabhängig von der schwer zu beantwortenden Frage, wie viele Bücher tatsächlich gelesen werden, zeigen die Ergebnisse aus der Fokusgruppenstudie und der Umfrage, dass E‑Books gedruckte Bücher vielmehr ergänzen, als dass sie diese ersetzen. Die heutigen Leser:innen können zwischen verschiedenen Lesemedien für den gleichen Text wählen bzw. diesen auch als Hörbuch rezipieren. So zeigen unsere Untersuchungen deutlich, dass Leser:innen von E‑Books grundsätzlich regelmäßige und vielseitige Buchleser:innen sind, die aufgrund pragmatischer Gründe zu digitalen Texten greifen. Als Motive für die Nutzung von E‑Books werden der Platzmangel in der eigenen Wohnung, die Mobilität und das geringe Gewicht digitaler Lesegeräte genannt sowie die Möglichkeit, jederzeit auf Online-Buchhandlungen oder die eigenen gespeicherten Bücher zugreifen zu können.

Aus historischer Sicht ist dies kein neues Phänomen, denn so führten bekanntlich die erweiterte Verfügbarkeit von Büchern (vor allem auch in Lesegesellschaften und Leihbibliotheken) sowie das Angebot kleinerer Formate im 18. Jahrhundert zu neuen Formen und neuen Qualitäten des Lesens. Gemeinsam mit der Säkularisierung des Buchangebots und der steigenden Alphabetisierung der Bevölkerung hat das zu neuen Lesepraktiken und dem geführt, was als Wende vom »intensiven« zum »extensiven Lesen« bezeichnet wird, d. h. vom mehrmaligen Lesen weniger Bücher zum einmaligen Lesen vieler Bücher.^55^

Die Transformation des gedruckten Buches hin zur leichter verfügbareren digitalen Version spricht jedenfalls eher Leser:innen an, die bereits viel lesen, als dass sie die Zahl der gelesenen Bücher grundsätzlich erhöht. Das bedeutet, dass vor allem erfahrene Buchleser:innen ihre bisherige Lesepraxis mit dem E‑Book erweitern, weil es den Bedürfnissen nach einer vielseitigen und rasch zur Verfügung stehenden Lektüre entspricht.

Betreffend der Lektüre-Auswahl nach Genres unterscheiden sich die Leser:innen, die sowohl gedruckte als auch digitale Bücher lesen (sogenannte Multi-Format-Leser:innen), von den reinen Print- oder Digital-Leser:innen. Sie weisen ein auffällig breites Spektrum an gelesenen Textsorten auf. Insgesamt bleibt aber auch bei ihnen das bevorzugte Format das gedruckte Buch. Aus leicht nachvollziehbaren Gründen werden lediglich erotische Romane bevorzugt digital gelesen: wegen der größeren Anonymität beim Kauf und der fehlenden paratextuellen Elemente, die Rückschlüsse auf den Inhalt des Textes durch andere erlauben könnten. Zusammenfassend lässt sich festhalten, dass das digitale Lesen von Büchern in der Freizeit vor allem von Personen genutzt wird, die viele Bücher, verschiedene Genres und darüber hinaus an unterschiedlichen Orten und in verschiedenen Situationen lesen. Das spezifische Materialitätsprofil und die spezifischen Affordanzen des E‑Books fördern somit eine Diversifizierung des Lesens von Literatur hinsichtlich der Menge und der Auswahl der Bücher. »Die perfekte Lesemaschine«, so Roland Reuß zur »Ergonomie des Buches«,^56^ und das bevorzugte Lesemedium bleibt für Viel-Leser:innen aber nach wie vor das gedruckte Buch, das nicht durch das digitale Pedant ersetzt wird, sondern dieses vielmehr aufgrund pragmatischer Motive und den Bedürfnissen nach rascher, vielseitiger und leicht zugänglicher Lektüre ergänzt.

### Leseorte und Lesesituationen

Während bis ins 18. Jahrhundert in Europa davon ausgegangen wurde, dass Bücher meist in Bibliotheken oder Studierstuben gelesen wurden, hat sich mit der Einführung des handlichen Oktavformats und der Verbreitung von Taschenbüchern ab den 1870er Jahren, vor allem aber im 20. Jahrhundert, eine Lesekultur entwickelt, die das Lesen mobilisierte.^57^ Es ist daher nicht überraschend, dass sich die Auswirkungen der jüngsten materialen Veränderung des Textträgers nun auch am eindeutigsten in veränderten Leseorten und neuen Lesesituationen zeigen. Im Vergleich zu Printleser:innen lesen Personen, die auch oder ausschließlich digital lesen, deutlich mehr, wenn sie nur wenig Zeit zum Lesen haben und sie sich in öffentlichen Räumen, wie in Verkehrsmitteln, an Plätzen im Freien und in öffentlichen Gebäuden sowie am Arbeitsplatz befinden. Besonders zeigt sich das in den Nutzungspraktiken derjenigen, die sowohl gedruckt als auch digital lesen und sich je nach Situation für ein Lesemedium entscheiden: Während das gedruckte Buch von ihnen zu Hause, tagsüber und bei ausreichender Zeit bevorzugt wird, ist das bevorzugte Lesemedium für die Lektüre in öffentlichen Verkehrsmitteln, an einem öffentlichen Ort, im Urlaub und wenn nur wenig Zeit zum Lesen bleibt, das digitale.

E‑Books verlagern somit das Lesen in Situationen, in denen das gedruckte Buch bisher nicht genutzt wurde und Leser:innen von E‑Books geben an, an neuen Orten zu lesen, seit sie mit dem digitalen Lesen begonnen haben. Wiederholte Unterbrechungen des Leseprozesses und ein fallweise oberflächliches Lesen auf elektronischen Lesegeräten sind aus dieser Sicht weniger durch die Materialität des Lesemediums selbst bestimmt, als vielmehr durch die Umstände und Situationen, in denen digitale Texte bevorzugt gelesen werden.

### Lesemodi und Lesepraktiken

Die Navigation durch digitale Texte wird immer wieder als nichtlineares Lesen beschrieben und mit Hypertexten oder multimodalen Texten assoziiert.^58^ Wird der Fokus nun nicht auf derart mehrfach verlinkte textuelle Gebilde gelegt, die prinzipiell multilinear organisiert sind und bei denen der Lesepfad durch die Lesenden individuell gestaltet werden kann, so zeigen sich allerdings andere Lesepraktiken. Die von uns in den Fokusgruppen befragten Leser:innen geben an, dass sie E‑Book-Versionen eines literarischen Textes überwiegend in einem fort und nur einmalig lesen, während sie gedruckte Bücher häufiger nicht-linear und wiederholt lesen. Sie beschreiben, dass sie in gedruckten Büchern öfter umblättern, nach bestimmten Passagen suchen, Absätze wiederholen und Bücher öfter schließen und wieder öffnen. Die Annahme, dass das Lesen von Gedrucktem im Unterschied zum Lesen am Bildschirm eine vollständige Lektüre von der ersten bis zur letzten Seite motiviere, findet nach Maye in der Mediengeschichte des Lesens keine Bestätigung und so befördere der Kodex im Unterschied zur Schriftrolle geradezu ein diskontinuierliches Lesen.^59^ Es ist bemerkenswert, dass diese Praktiken, die einst mit der Einführung des Kodex möglich wurden, auch heute noch geschätzt werden.^60^

Die lineare Lektüre von E‑Books hängt auch damit zusammen, dass diese den Teilnehmer:innen unserer Fokusgruppen zufolge vor allem für Unterhaltungs- und Spannungsliteratur bevorzugt werden. Hoch geschätzte und geliebte Bücher sollen hingegen physisch im Besitz sein und bedingen dadurch auch andere Lesepraktiken, denn die Leser:innen möchten öfter darin blättern oder sie – wenigstens ausschnittweise – wiederholt lesen können. María Angélica Thumala Olave verweist darauf, dass die Vorstellung des Buches als Kodex, als ein in sich geschlossenes Objekt, das man besitzen kann, immer noch eine große Bedeutung hat, während die E‑Book-Lektüre tendenziell als einmaliges Erlebnis verstanden wird: »E-books are less possessions than temporary experiences.«^61^ Zusätzlich erhöhen das Verbleiben im Bücherregal und die dadurch bedingte Sichtbarkeit im Alltag die Wahrscheinlichkeit, dass ein gedrucktes Buch, das einmal nicht zu Ende gelesen wurde, zu Ende gelesen oder erneut gelesen wird. Die durch die spezifische Lesemotivation und das gewählte Genre indizierten Lesepraktiken beeinflussen also eher die Entscheidung für das Lesemedium, als dass ein bestimmtes Lesemedium konkrete Lesemodi befördere. Es kann davon ausgegangen werden, dass Leser:innen bereits vor Beginn der Lektüre eines Textes wissen, wie sie diesen lesen wollen und werden. Hier zeigt sich das reziproke Verhältnis zwischen schriftragendem Artefakt und Körper, sprich den Lesenden, denn wenn auch die Materialität der Artefakte bestimmte Praktiken nicht determiniert, so beeinflusst sie doch das Spektrum der Möglichkeiten des Buchgebrauchs.^62^

### Buchbesitz und Literatur als Artefakt

In den Fokusgruppeninterviews berichten E‑Book-Leser:innen, dass sie wiederholt gedruckte Ausgaben von Büchern kaufen, die sie zuvor bereits in digitaler Form gelesen haben, um sie wirklich zu »besitzen«.^63^ Geliebte und geschätzte literarische Texte werden von E‑Book-Leser:innen aber nicht aufgrund einer womöglich besseren Leseerfahrung zusätzlich in gedruckter Form erworben, sondern aufgrund der ikonischen Funktion des Buches als symbolisches Objekt. E‑Books sind praktische und für den einmaligen Gebrauch vorgesehene Gegenstände, während gedruckte Bücher als individuelle Objekte von dauerhaftem Wert gesehen werden. Damit wird erkennbar, dass die Bedeutung des Mediums Buch nicht auf seine Funktion im System der Medienkommunikation beschränkt bleibt. Als materiell fassbare Gegenstände können gedruckte Bücher »gezeigt, benutzt und in symbolische Handlungen oder Inszenierungen der Lebenswelt eingebettet werden.«^64^ Die inhaltliche und ästhetische Individualität des gedruckten Buches wird durch den spezifischen Einband, das Papier, das Layout, die Schriftgröße und die Schriftart materiell fassbar, während bei E‑Books diese paratextuellen Merkmale bei jedem Text mehr oder weniger gleich bleiben bzw. nach Präferenz der Leser:innen angepasst werden können. Daher eignen sich gedruckte Bücher in besonderer Weise dazu, »den eigenen Geschmack, die eigene Bildung und geistige Haltung, ja die gesamte soziale Identität auszudrücken«^65^, während E‑Books aufgrund der fehlenden individuellen Präsenz diese, vor allem nach der Lektüre zum Tragen kommende, Funktion nicht aufweisen.

Aus unseren Interviews geht hervor, dass es entgegen verbreiteter Annahmen weniger die Haptik, das Papier, das Hantieren mit den Seiten im Leseprozess sind, die dem gedruckten Buch den Vorzug geben, als vielmehr das Buch in seiner ganzen individuellen Materialität und damit als Träger eines bestimmten Inhalts in einer konkreten Lesesituation. Jedes einzelne Papierbuch hat notwendigerweise einen eigenen und deutlich erkennbaren Ort, denn »ein Text ist in der Form des gedruckten Buches eine von anderen Texten deutlich abgegrenzte Einheit.«^66^ Und damit ist es nur plausibel, dass Leser:innen wiederholt angeben, dass die beim Lesen gemachten emotionalen und intellektuellen Erfahrungen und teilweise auch die spezifische Lesesituation beim Anblick eines konkreten Buches wieder hervorgerufen werden. Gedruckte Bücher besitzen somit eine Funktion als materielle Artefakte, welche E‑Books auf digitalen Endgeräten, die als austauschbare Lesegräte fungieren, nicht erfüllen können.

## Fazit

Das zentrale Ergebnis unserer empirischen Untersuchungen zur Frage nach den Konsequenzen des Lesens digitalisierter Literatur im Vergleich zu gedruckten Büchern besteht zusammengefasst darin, dass wir die wesentlichen Veränderungen nicht im konkreten Akt des Lesens, dem Textverstehen oder der literarischen Erfahrung feststellen konnten, sondern in den Lese- und Nutzungspraktiken von Büchern. Sowohl was die Menge des Gelesenen betrifft als auch die Lektüreauswahl, die Leseorte und Lesesituationen sowie die Erwerbs- und Aufbewahrungsformen, münden die unterschiedlichen Materialitätsprofile von digitalisierten und gedruckten Büchern in unterschiedliche Praxeographien. Allerdings erfüllen beide Lesemedien unterschiedliche Funktionen, gehen mit verschiedenen Lesepraktiken einher und ergänzen sich vielmehr, als sich zu ersetzen. Denn bei den meisten Leser:innen geht es nicht um die Frage der Verdrängung des alten Mediums durch ein neues, sondern um die bewusste Wahl des geeigneteren in bestimmten Lesesituationen.

In der zukünftigen Forschung wäre insbesondere die Frage nach konkreten Leser:innentypen, Lesemotivationen und den spezifischen Funktionen verschiedener Lesemedien zu verfolgen, ebenso die Frage nach dem Einfluss bestimmter gesellschaftlicher Milieus und Bildungsverhältnisse auf die Nutzungspraktiken, den symbolischen Wert des gedruckten Buches und die Text- und Medienauswahl. Eine Forschungslücke besteht nach wie vor in der Untersuchung der Auswirkungen verschiedener Leseapplikationen und Leseplattformen sowie dadurch bestimmte Praktiken der Annotation und Interaktion auf den Umgang mit literarischen Texten. Vor allem die Lesepraktiken auf Fanfiction-Plattformen, auf denen literarische Texte nicht nur gelesen, sondern auch geschrieben und kommentiert werden, gilt es als besondere und ausschließlich digital stattfindende Form des Lesens genauer zu bestimmen. Methodisch ebenso relevant wie herausfordernd ist eine detailliertere Untersuchung konkreter literarischer Strukturen und Genrespezifika sowie die Frage, ob Texte mit einem hohen literarischen Komplexitätsgrad in verschiedenen Medien nicht doch zu unterschiedlicher Wirkung gelangen. Zielführend erscheint die Verknüpfung einer dezidierten Perspektive auf textinhärente Strukturmerkmale, die über die Unterscheidung zwischen informativen und narrativen Texten hinausgeht, mit einer praxeologischen Betrachtung der unterschiedlichen digitalen Lesemedien im Vergleich.

## Footnotes

1 Debray, Régis: *Einführung in die Mediologie*. Bern/Stuttgart/Wien: Haupt Verlag, 2003, S. 54 f.

2 Chartier, Roger: *Lesewelten. Buch und Lektüre in der frühen Neuzeit*. Frankfurt a.M.: Campus Verlag, 1990, S. 7 f.

3 Vgl. Kuhn, Axel/Hagenhoff, Svenja: »Kommunikative statt objektzentrierte Gestaltung: Zur Notwendigkeit veränderter Lesekonzepte und Leseforschung für digitale Lesemedien«. In: Sebastian Böck/Julian Ingelmann/Friederike Schruhl/Kai Matuszkiewicz (Hg.): *Lesen X.0 Rezeptionsprozesse in der digitalen Gegenwart*. Göttingen: V&R unipress, 2017, S. 27–45. 10.14220/9783737007450.27, S. 28.

4 Maye, Harun: »Medien des Lesens«. In: Rolf Parr/Alexander Honold (Hg.): *Grundthemen der Literaturwissenschaft: Lesen*. Berlin/Boston: De Gruyter, 2018, S. 103–122. 10.1515/9783110365252-003, S. 105.

5 Griem, Julika: *Szenen des Lesens. Schauplätze einer gesellschaftlichen Selbstverständigung*. Bielefeld: transcript, 2021, S. 28.

6 Z. B. Carr, Nicholas: *The Shallows: What the Internet Is Doing to Our Brains*. New York: W. W. Norton & Company, 2010; Wolf, Maryanne: *Reader, come home: The reading brain in a digital world. *New York: Harper Collins, 2018.

7 Z. B. Baron, Naomi S.: *Words Onscreen: The Fate of Reading in a Digital World*. New York: Oxford University Press, 2015.

8 Lauer, Gerhard: *Lesen im digitalen Zeitalter*. Darmstadt: wbg Academic, 2020, S. 11.

9 Z. B. die Special Issues: Ashton, Dan/Bower, Gavin James/Hollyman, Steve/Kate Pullinger, Kate (Hg.): »Writing Digital: Practice, Performance, Theory.« In: *Convergence *23/1 (2017). 10.1177/1354856516679755; Hjarvard, Stig/Helles, Rasmus (Hg.): »Books and Publishing in a Digital Age.« In: *Northern Lights *13/1 (2015). 10.1386/nl.13.1.3_7.

10 Vgl. Andersen, Tore R./Kjerkegaard, Stefan/Pedersen, Birgitte S.: »Introduction: Modes of Reading«. In: *Poetics Today* 42/2 (2021), S. 131–147. 10.1215/03335372-8883164, S. 134.

11 Vgl. Baron, Naomi S.: *How We Read Now: Strategic Choices for Print, Screen, and Audio*. New York: Oxford University Press, 2021.

12 Honold, Alexander/Parr, Rolf: »Einleitung. Lesen – literatur-, kultur- und medienwissenschaftlich«. In: Dies.: *Grundthemen der Literaturwissenschaft: Lesen*. Berlin/Boston: De Gruyter, 2018, S. 3–26. 10.1515/9783110365252-001, S. 20.

13 Penke, Niels/Werber, Niels: »Medien der Literatur. Zur Einleitung«. In: *Zeitschrift für Literaturwissenschaft und Linguistik* 49 (2019), S. 369–373. 10.1007/s41244-019-00145-z, S. 371.

14 Vgl. Schwabe, Annika/Brandl, Lukas/Boomgaarden, Hajo G./Stocker, Günther: »Experiencing literature on the e‑reader: The effects of narrative texts on screen«. In: *Journal of Research in Reading* 44/2 (2021), S. 319–338. 10.1111/1467-9817.12337; Kosch, Lukas/Stocker, Günther/Schwabe, Annika/Boomgaarden, Hajo G.: »Reading fiction with an e‑book or in print: Purposes, pragmatics and practices. A focus group study«. In: *Scientific Study of Literature* 11/2 (2022), S. 196–222. 10.1075/ssol.21012.kos; Schwabe, Annika/Kosch, Lukas/Boomgaarden, Hajo G./Stocker, Günther: »Book Readers in the Digital Age: Reading Practices and Media Technologies«. In: *Mobile Media & Communication* (2022). 10.1177/20501579221122208.

15 Vgl. Couldry, Nick: »Theorising media as practice«. In: Birgit Bräuchler/John Postill (Hg.): *Theorising media and practice*. Oxford/New York: Berghan Books, 2010, S. 35–54.

16 Reckwitz, Andreas: »Der Ort des Materiellen in den Kulturtheorien. Von sozialen Strukturen zu Artefakten«. In: Ders.: *Unscharfe Grenzen: Perspektiven der Kultursoziologie*. Bielefeld: transcript, 2. unveränderte Auflage 2010, S. 131–156. 10.1515/9783839409176-00, S. 147.

17 Reckwitz 2010, S. 154. In seinen »Grundelementen einer Theorie sozialer Praktiken« bezieht sich Reckwitz dann auch explizit auf das Lesen, indem er ›Praktik‹ wie folgt definiert: »Eine Praktik *besteht* aus bestimmten routinisierten Bewegungen und Aktivitäten des Körpers. Dies gilt ebenso für intellektuell ›anspruchsvolle‹ Tätigkeiten wie die des Lesens, Schreibens oder Sprechens.« Reckwitz, Andreas: »Grundelemente einer Theorie sozialer Praktiken«. In: *Zeitschrift für Soziologie* 32/4 (2003), S. 282–301, S. 290.

18 Vgl. Hilgert, Markus: »›Text-Anthropologie‹. Die Erforschung von Materialität und Präsenz des Geschriebenen als hermeneutische Strategie«. In: Ders. (Hg.): *Altorientalistik im 21. Jahrhundert. Selbstverständnis, Herausforderungen, Ziele*. Mitteilungen der Deutschen Orientgesellschaft 142 (2010), S. 87–126.

19 Hilgert, Markus: »Praxeologisch perspektivierte Artefaktanalysen des Geschriebenen: Zum heuristischen Potential der materialen Textkulturforschung«. In: Friederike Elias/Albrecht Franz/Henning Murmann/Ulrich Wilhelm Weiser: *Praxeologie: Beiträge zur interdisziplinären Reichweite praxistheoretischer Ansätze in den Geistes- und Sozialwissenschaften*. Berlin/München/Boston: De Gruyter, 2014, S. 149–164. 10.1515/9783110370188.149, S. 162.

20 Hilgert 2014, S. 162.

21 Vgl. Maye 2018, S. 115.

22 Vgl. Stocker, Günther: »›Aufgewacht aus tiefem Lesen.‹ Überlegungen zur Medialität des Bücherlesens im digitalen Zeitalter«. In: Hans-Christian von Herrmann/Jeannie Moser (Hg.): *Lesen. Ein Handapparat*. Frankfurt a.M.: Vittorio Klostermann, 2015, S. 33–48, S. 37.

23 Vgl. Schilhab, Theresa/Balling, Gitte/Kuzmičová, Anežka: »Decreasing materiality from print to screen reading«. In: *First Monday* 23/10 (2018). 10.5210/fm.v23i10.9435.

24 Maye 2018, 105 f.

25 Vgl. Mangen, Anne: »Hypertext fiction reading: haptics and immersion«. In: *Journal of Research in Reading* 31 (2008), S. 404-419. 10.1111/j.1467-9817.2008.00380.x.

26 Reuß, Roland: *Die perfekte Lesemaschine. Zur Ergonomie des Buches*. Göttingen: Wallstein, 2. Auflage 2016, S. 14.

27 Reuß 2016, S. 10.

28 Reuß 2016, S. 11.

29 Die Aufmerksamkeit für die medialen Spezifika bedingt auch eine exakte Unterscheidung zwischen verschiedenen digitalen Lesegeräten für E‑Books wie dem in der Gestaltung dem gedruckten Buch nachempfundenen, monofunktionalen und wenig interaktiven E‑Reader, dem Smartphone mit seinem vergleichsweise kleinen Bildschirm, dem Tablet und dem Notebook. Darüber hinaus stellen verschiedene Apps und Softwareprogramme unterschiedliche Möglichkeiten der Interaktion mit Texten zur Verfügung.

30 Gibson, James J.: *Wahrnehmung und Umwelt: der ökologische Ansatz in der visuellen Wahrnehmung*. München/Wien: Urban & Schwarzenberg, 1982.

31 Vgl. Fox, Richard/Panagiotopoulos, Diamantis/Tsouparopoulou, Christina: »Affordanz«. In: Thomas Meier/Michael R. Ott/Rebecca Sauer (Hg.): *Materiale Textkulturen: Konzepte – Materialien – Praktiken*. Berlin/München/Boston: De Gruyter, 2015, S. 63–70. 10.1515/9783110371291.63.

32 Vgl. Focken, Friedrich-Emanuel/Elias, Friederike/Witschel, Christian/Meier, Thomas: »Material(itäts)profil – Topologie – Praxeographie«. In: Thomas Meier/Michael R. Ott/Rebecca Sauer (Hg.): *Materiale Textkulturen: Konzepte - Materialien – Praktiken*. Berlin/München/Boston: De Gruyter, 2015, S. 129–134. 10.1515/9783110371291.129, S. 133.

33 Mangen, Anne/van der Weel, Adriaan: »The evolution of reading in the age of digitisation: an integrative framework for reading research«. In: *Literacy* 50 (2016), S. 116-124. 10.1111/lit.12086, S. 121.

34 Maye 2018, S. 106.

35 Vgl. dazu Reuß: »Daß das Buch ein für sich seiender, konkreter, dreidimensionaler Gegenstand ist, erscheint aus der kyklopischen Perspektive von ›Netz‹-Publikationen als Besonderheit, als Auszeichnungsmerkmal. Es kann angefaßt werden, liegt ›in der *Hand*‹. Finger eines Menschen halten es beim Lesen, wenden die Blätter, können mit einem anderen Gegenstand, einem Stift, an seinen Rand schreiben. Der deutsche Ausdruck ›Begreifen‹, der zwischen einer übertragenen und einer konkreten Bedeutung schwankt, entspricht wie sein genaues Pendant – das ›Erfassen‹ – der materiellen Objektivität des Buches.« Reuß 2016, S. 39.

36 Vgl. Focken/Elias/Witschel/Meier 2015, S. 133.

37 Vgl. Burke, Michael: *Literary Reading, Cognition and Emotion: An Exploration of the Oceanic Mind*. New York: Routledge, 2010. 10.4324/9780203840306, S. 94.

38 Vgl. Clinton, Virginia: »Reading from paper compared to screens: A systematic review and meta-analysis«. In: *Journal of Research in Reading* 42/2 (2019), S. 288–325. 10.1111/1467-9817.12269; Delgado, Pablo/Vargas, Cristina/Ackerman, Rakefet/Salmerón, Ladislao: »Don’t throw away your printed books: A meta-analysis on the effects of reading media on reading comprehension«. In: *Educational Research Review* 25 (2018), S. 23–38. 10.1016/j.edurev.2018.09.003.

39 Vgl. Schwabe, Annika/Brandl, Lukas/Boomgaarden, Hajo G./Stocker, Günther: »Literatur am Bildschirm – Zum Stand der empirischen Leseforschung«. In: *Orbis Litterarum* 75 (2020), S. 213–229. 10.1111/oli.12271.

40 Geiger, Arno: *Schöne Freunde*. München: Hanser, 2002.

41 Appel, Markus/Koch, Erik/Schreier, Margrit/Groeben, Norbert: »Aspekte des Leseerlebens: Skalenentwicklung«. In: *Zeitschrift für Medienpsychologie* 14/4 (2002), S. 149–154. 10.1026//1617-6383.14.4.149.

42 Koopman, Eva Maria E.: »How texts about suffering trigger reflection: genre, personal factors, and affective responses«. In: *Psychology of Aesthetics, Creativity, and the Arts* 9/4 (2015), S. 430–441. 10.1037/aca0000006.

43 Detaillierte Angaben zum Verfahren, zur Datenanalyse und allen Teilergebnissen inkl. Tabellen zu Effektstärken etc. finden sich in Schwabe/Brandl/Boomgaarden/Stocker 2021.

44 Die Detailergebnisse finden sich in Kosch/Stocker/Schwabe/Boomgaarden 2022.

45 Die Umfrage wurde in Kooperation mit dem Markt- und Meinungsforschungsinstitut marketagent.com durchgeführt.

46 Die Detailergebnisse finden sich in Schwabe/Kosch/Boomgaarden/Stocker 2022.

47 Krämer, Sybille: »Epistemologie der Medialität: Eine medienphilosophische Reflexion«. In: *Deutsche Zeitschrift für Philosophie* 67/5 (2019), S. 833–850. 10.1515/dzph-2019-0061, S. 834.

48 Kalthoff, Herbert/Cress, Torsten/Röhl, Tobias: »Einleitung: Materialität in Kultur und Gesellschaft«. In: Dies.: *Materialität. Herausforderungen für die Sozial- und Kulturwissenschaften*. Leiden: Brill/Fink, 2016, S. 11–41. 10.30965/9783846757048_003, S. 27.

49 Bäcker, Iris: *Der Akt des Lesens – neu gelesen. Zur Bestimmung des Wirkungspotenzials von Literatur*. München: Fink, 2014, S. 37.

50 Vgl. Mersch, Dieter: »Wort, Bild, Ton, Zahl: Eine Einleitung in die Medienphilosophien.«. In: Ders.: *Kunst und Medium. Zwei Vorlesungen. *Gestalt und Diskurs Bd. III. Hg. v. Theresa Georgen. Kiel: Muthesius Hochschule, 2002, S. 131–254, S. 135.

51 Vgl. Zwaan, Rolf A.: *Aspects of Literary Comprehension. A Cognitive Approach*. Amsterdam/Philadelphia: John Benjamins, 1993.

52 Vgl. dazu auch Altmann, Ulrike/Bohrn, Isabel/Lubrich, Oliver/Menninghaus, Winfried/Jacobs, Arthur M.: »Fact vs fiction – how paratextual information shapes our reading processes«. In: *Social cognitive and affective neuroscience *9/1 (2012), S. 22–29. 10.1093/scan/nss098.

53 Vgl. dazu auch Rosebrock, Cornelia: »Strategien ästhetischen Lesens. Literarisches Lernen in rezeptionsästhetischer Perspektive«. In: Daniel Scherf/Andrea Bertschi-Kaufmann (Hg.): *Ästhetische Rezeptionsprozesse in Didaktischer Perspektive*. Weinheim: Beltz Juventa, 2018, S. 14–27.

54 Lauer 2020, S. 42.

55 Vgl. z. B. Engelsing, Rolf: *Der Bürger als Leser. *Stuttgart: Metzler, 1974; Wittmann, Reinhard: *Geschichte des deutschen Buchhandels*. München: C.H. Beck, 2010.

56 Reuß 2016.

57 Vgl. Wittmann 2010. Literaturhistorisch präzise formuliert ist das auch in Goethes Werther, dessen Titelheld die Mobilität seiner kleinen »Wetsteinischen« Homer-Ausgabe im Duodezformat lobt, die es erst möglich macht, »seinen Homer« auf seinen Spaziergängen mitzunehmen und in der freien Natur zu lesen: Goethe, Johann Wolfgang: »Die Leiden des jungen Werthers«. In: Ders.: *Sämtliche Werke*. Band 8. Hg. v. Waltraud Wiethölter. Frankfurt a.M.: Deutscher Klassiker Verlag, 1994, S. 10–268. S, 110.

58 Vgl. z. B. Philipp, Maik: *Multiple Dokumente verstehen. Theoretische und empirische Perspektiven auf Prozesse und Produkte des Lesens mehrerer Dokumente*. Weinheim: Beltz, 2020; Rosebrock, Cornelia: »Netzlektüre und Deep Reading: Entmischungen der Lesekultur«. *Leseforum Schweiz 2* (2020), S. 1–16.

59 Maye 2018, S. 117.

60 Vgl. Mole, Tom: *The Secret Life of Books. Why They Mean More Than Words*. London: Elliott and Thompson, 2019.

61 Thumala Olave, María A.: »Book love. A cultural sociological interpretation of the attachment to books«. In: *Poetics* 81 (2020). 10.1016/j.poetic.2020.101440, S. 7.

62 Vgl. Reckwitz 2010, S. 154.

63 Tatsächlich bedeutet der Kauf eines E‑Books nicht den Übergang ins Eigentum, denn »ungeachtet der bestehenden Unklarheit werden digitale Inhalte im Internet massenhaft mit dem Schlagwort des ›Kauf‹ angepriesen, obgleich der daraufhin abgeschlossene Vertrag laut Allgemeiner Geschäftsbedingungen lediglich Nutzungsrechte einräumt.« Lafer, Rosa: *Der digitale Erwerb eines E‑Books: Eine vertragstypologische Untersuchung auf der Schnittstelle zwischen Nutzungsrechteinräumung und Bereitstellung eines immateriellen Gegenstands*. Baden-Baden: Nomos, 2020, S. 21.

64 Rautenberg, Ursula: »Das Buch in der Alltagskultur: Eine Annäherung an zeichenhaften Buchgebrauch und die Medialität des Buches«. In: *Alles Buch. Studien der Erlanger Buchwissenschaft XV* 2005, S. 5.

65 Schneider, Ute: »Bücher zeigen und Leseatmosphäre inszenieren – vom Habitus enthusiastischer Leserinnen und Leser«. In: Steffen Martus/Carlos Spoerhase (Hg.): *Gelesene Literatur – Populäre Lektüre im Medienwandel*. München (edition text + kritik. Sonderband 12) 2018, S. 113–123, S. 115.

66 Stocker 2015, S. 39.
